# Modelling the prevalence of hepatitis B towards eliminating it as a major public health threat in China

**DOI:** 10.1186/s12889-022-13594-y

**Published:** 2022-06-13

**Authors:** Wenjun Liu, Tianyi Zhuang, Ruyi Xia, Zhuoru Zou, Lei Zhang, Mingwang Shen, Guihua Zhuang

**Affiliations:** 1 Department of Epidemiology and Biostatistics, School of Public Health, Xi’an Jiaotong University Health Science Center, Xi’an, 710061 Shaanxi China; 2 Xi’an Center for Disease Control and Prevention, Xi’an, Shaanxi China; 3 China-Australia Joint Research Centre for Infectious Diseases, School of Public Health, Xi’an Jiaotong University Health Science Centre, Xi’an, Shaanxi China; 4 Melbourne Sexual Health Centre, Alfred Health, Melbourne, VIC Australia; 5 Key Laboratory for Disease Prevention and Control and Health Promotion of Shaanxi Province, Xi’an, Shaanxi China

**Keywords:** Hepatitis B, Prevalence, Mathematical model, Prediction, Peripartum antiviral prophylaxis

## Abstract

**Background:**

The World Health Organization (WHO) requires reduction in the prevalence of hepatitis B virus (HBV) surface antigen (HBsAg) in children to 0.1% by 2030, a key indicator for eliminating viral hepatitis as a major public health threat. Whether and how China can achieve this target remains unknown, although great achievements have been made. We aimed to predict the decline of HBsAg prevalence in China and identify key developments needed to achieve the target.

**Methods:**

An age- and time-dependent dynamic compartmental model was constructed based on the natural history of HBV infection and the national history and current status of hepatitis B control. The model was run from 2006 to 2040 to predict the decline of HBsAg prevalence under three scenarios including maintaining current interventions (status quo), status quo + peripartum antiviral prophylaxis (PAP, recommended by WHO in 2020), and scaling up current interventions + PAP.

**Results:**

Under the status quo, HBsAg prevalence would decrease steadily in all age groups, but the WHO’s target of 0.1% prevalence in children aged < 5 years would not be achieved until 2037. The results are robust according to sensitivity analyses. Under the status quo + PAP, the HBsAg prevalence of children aged < 5 years would significantly decrease with the introduction of PAP, and the higher the successful interruption coverage is achieved by PAP, the more significant the decline. However, even if the successful interruption coverage reaches 90% by 2030, the 0.1% prevalence target would not be met until 2031. Under the scaling up current interventions + PAP, combined with scale-up of current interventions, the WHO’s 0.1% target would be achieved on time or one year in advance if PAP is introduced and the successful interruption coverage is scaled up to 80% or 90% by 2030, respectively.

**Conclusions:**

It is difficult for China to achieve the WHO’s target of 0.1% HBsAg prevalence in children by 2030 by maintaining current interventions. PAP may play an important role to shorten the time to achieve the target. A comprehensive scale-up of available interventions including PAP will ensure that China achieves the target on schedule.

**Supplementary Information:**

The online version contains supplementary material available at 10.1186/s12889-022-13594-y.

## Background

Hepatitis B virus (HBV) infection has long been a major health problem in China. The first two national hepatitis B serosurveys in 1979 and 1992, respectively, showed that 9.05% and 9.75% of Chinese aged 1 − 59 years were positive for HBV surface antigen (HBsAg) [1]. In 1992, the Chinese government recommended routine hepatitis B vaccination for newborns, but with a policy of “self-select and self-pay”. This strategy was integrated into the National Children Immunization Program in 2002 with a free policy, which led to a rapid increase in the vaccine coverage of newborns. In 2006, the third national serosurvey showed that the HBsAg prevalence of Chinese aged 1 − 59 years had dropped to 7.18%. More importantly, significant declines had occurred in children aged 1 − 4 and 5 − 14 years, from 9.67% and 10.74% in 1992 to 0.96% and 2.42% in 2006, respectively [2]. The achievement prompted the Chinese government to carry out a catch-up vaccination campaign during 2009 − 2011 for children born between 1994 − 2001 who missed the routine vaccination. The latest national serosurvey in 2014, in which only people aged 1 − 29 years were enrolled, showed that the HBsAg prevalence of children aged 1 − 4 and 5 − 14 years further dropped to 0.32% and 0.94%, respectively [3]. The experience from China confirms that newborn vaccination is the most crucial measure to control hepatitis B in highly HBV-endemic countries [4–6]. Despite significant gains made during the past 30 years, China still maintains a high HBsAg prevalence (5 − 6% in total population) and the largest burden of chronic HBV carriers (estimated 70 million) in the world [7].

In 2016, the World Health Organization (WHO) released the first global health sector strategy on viral hepatitis for contributing to the achievement of the 2030 Agenda for Sustainable Development. The strategy outlined a way ahead, and provided a goal towards eliminating viral hepatitis as a major public health threat by 2030. For hepatitis B, reducing new chronic infections by 90% was required as a key impact target, which is equivalent to reducing HBsAg prevalence among children under 5 years to 0.1% [8, 9]. One previous study has addressed the global issue and concluded that the target could be achieved by scale-up of vaccine coverage in newborns and innovations in scalable options for prevention of mother-to-child transmission [10]. However, China has its own characteristics, especially high HBsAg prevalence in women of childbearing age and high newborn vaccine coverage which has been reached. Whether and how China can achieve the target requires specifically targeted studies, which will contribute to the achievement of the global goal. This study aimed to predict the decline of HBsAg prevalence in China by using a mathematical model and identify key developments needed to achieve the target. The study finding will inform policy-makers to improve intervention strategies and programs further.

## Methods

### Model construction

**Fig. 1:**
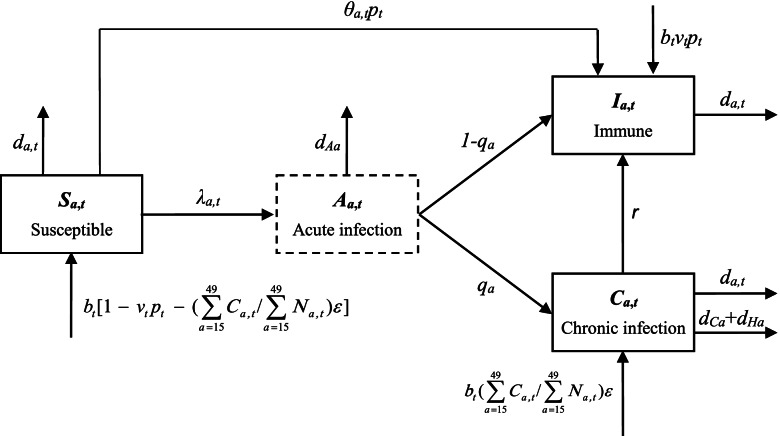
Age- and time-dependent dynamic compartmental model of HBV transmission. Boxes with solid line represent compartments of the transmission process, and lines with arrowhead represent transitions and their directions. The box with dashed line is not a compartment, only representing the transient process of acute HBV infection. *λ*_*a,t*_, age- and time-dependent force of HBV infection; *q*_*a*_, age-dependent proportion of acute HBV infections that become chronic; *r*, rate of chronic HBV infections that become immune (HBV clearance); *d*_*a,t*_, age- and time-dependent background mortality; *d*_*Aa*_, age-dependent mortality of acute HBV infection; *d*_*Ca*_, age-dependent mortality of cirrhosis; *d*_*Ha*_, age-dependent mortality of hepatocellular carcinoma; *v*_*t*_, vaccine coverage of newborns at a given time; *p*_*t*_, vaccine protection against HBV infection at a given time; *θ*_*a,t*_, catch-up vaccine coverage during 2009 − 2011 in children born between 1994 − 2001 who missed the routine vaccination; *b*_*t*_, birth rate at a given time; *ɛ*, HBV intrauterine infection rate in infected pregnant women; *N*_*a,t*_, total number of people with a specific age at a given time. $\sum\limits_{a = 15}^{49} {C_{a,t} } /\sum\limits_{a = 15}^{49} {N_{a,t} }$ denotes HBV carriage rate of women with childbearing age (15 − 49 years) at a given time

We constructed an age- and time-dependent dynamic compartmental model by extending our previous model [5], to simulate HBV transmission in China (Fig. 1), based on the natural history of HBV infection and the national history and current status of hepatitis B control. The population was divided into three compartments including susceptible to HBV (*S*_*a,t*_), immune due to infection or vaccination (*I*_*a,t*_), and chronic infection (*C*_*a,t*_), in which *a* and *t* represent the age and the time, respectively. Acute infection (*A*_*a,t*_) is not a compartment but rather a transient process by which a susceptible person moves to other compartments or dies. The population was further divided into 101 age groups, one for each age, from 0 to 100 years. The model was run annually, which means with one transition occurring, the age of all individuals increases by one year and a cohort of newborns enter the population. The relevant discrete difference equations were shown below (Eqs. (1) and (2)).

For individuals aged 0 year:

1 $$\left\{ \begin{gathered} S_{0,t} = b_{t} N_{t} [1 - v_{t} p_{t} - (\sum\limits_{a = 15}^{49} {C_{a,t} } /\sum\limits_{a = 15}^{49} {N_{a,t} } )\varepsilon ] \hfill \\ \hfill \\ C_{0,t} = b_{t} N_{t} (\sum\limits_{a = 15}^{49} {C_{a,t} } /\sum\limits_{a = 15}^{49} {N_{a,t} } )\varepsilon \hfill \\ \hfill \\ I_{0,t} = b_{t} N_{t} v_{t} p_{t} \hfill \\ \end{gathered} \right.$$

For individuals aged 1 − 100 years:

2 $$\left\{\begin{array}{c}{S}_{a+1,t+1}={S}_{a,t}\left(1-{\lambda }_{a,t}-{d}_{a,t}-{\theta }_{a,t}{p}_{t}\right)\\ {C}_{a+1,t+1}={C}_{a,t}\left(1-{d}_{a,t}-{d}_{Ca}-{d}_{Ha}-r\right)+{S}_{a,t}{\lambda }_{a,t}\left(1-{d}_{Aa}\right){q}_{a}\\ {I}_{a+1,t+1}={I}_{a,t}\left(1-{d}_{a,t}\right)+{S}_{a,t}{\lambda }_{a,t}\left(1-{d}_{Aa}\right)\left(1-{q}_{a}\right)+{S}_{a,t}{\theta }_{a,t}{p}_{t}+{C}_{a,t}r\end{array}\right.$$

where $$N_{a,t} = S_{a,t} + C_{a,t} + I_{a,t}$$, and $$N_{t} = \sum\limits_{a = 0}^{100} {N_{a,t} }$$.

### The force of HBV infection

The force of infection (*λ*) is defined as the probability per unit of time that a susceptible person becomes infected and, theoretically, it can be written as follows [11]:

3 $$\lambda = k\beta \frac{C}{N} = \phi \frac{C}{N}$$

where *C* is the number of infectious individuals, *N* the total number of individuals in the population, *k* the average number of contacts made by an infectious individual, and *β* the probability of transmission following a contact between infectious and susceptible individuals. *φ* is the product of *k* and *β*, which is called the transmission coefficient. Vaccination can directly impact the proportion of infectious individuals in a population (*C/N*), so it affects the transmission of pathogen in the population. However, vaccination by itself cannot directly impact the variable *k* or *β*, or the product of both *φ*. The change of *φ* depends on other factors, for hepatitis B, including safe injection practice, blood donation screening, management and treatment of chronic HBV-infected persons, and other non-immunization factors.

Due to the incomplete information and poor quality of reported acute hepatitis B incidence data in China, we estimated the age- and time-dependent force of HBV infection (*λ*_*a,t*_) based on the previous national hepatitis B serosurvey data (see Supplementary Materials for details). First, by a modified simple catalytic model we estimated the age-dependent force of HBV infection in 1992 (*λ*_*a,1992*_) from the 1992 serosurvey data (Table S1). Second, we built a matrix of “who acquires infection from whom” (Figure S1) and modified Eq. (3) to Equation (S1) according to the matrix to calculate the corresponding transmission coefficients in 1992 (Table S2). Third, we selected an exponential function to characterize the decline of transmission coefficients from 1992 to 2006 and used Markov Chain Monte Carlo method with a Metropolis–Hastings algorithm to estimate parameters of the function, i.e. decline curves of transmission coefficients with time (Figure S2), in which the 2006 serosurvey data were used as calibrations of the model outputs (Figure S3). Finally, the 2014 serosurvey data were used to validate our model (Figure S4). By this process, the declining pattern of transmission coefficients over time was identified, which is associated with non-immunization interventions. The declining pattern was maintained throughout our prediction analysis.

### Other model parameters

The model was run from 2006 to 2040 to predict the decline of HBsAg prevalence. Initial conditions of the model were determined according to the 2006 serosurvey data and China Population and Employment Statistics Yearbook, 2007. Because no people aged > 59 years were enrolled in the serosurvey, the HBV test data of people aged 55 − 59 years were used for the elderly. Birth rates and age-specific background mortalities during 2006 − 2020 were obtained from the corresponding yearbooks, but after 2020, they were assumed to follow the 2020 data. For birth rates, an annual change by ± 5% was introduced after 2020 to cover its uncertainty given changes of the family planning policy in China.

The full three-dose vaccine coverage and the birth dose vaccine coverage for newborns have stabilized at high levels at national-level over the last decade in China, according to reports from the National Immunization Information System [12, 13]. At the same time, hepatitis B immunoglobulin (HBIG) has also been promoted for use in newborns of HBsAg-positive mothers [7]. However, reported coverages varied substantially in province-level, especially in county-level (from < 90% to 99%), significantly lower in rural counties [12, 13]. In addition, overstated reports are also constantly concerned [12–15]. Based on these situations, the base-case value of vaccine coverage of newborns was estimated at 94% in 2006 and beyond, which is close to the work objective (95%) of the China Viral Hepatitis Prevention and Treatment Plan (2017 − 2020), and a wide range (90% to 98%) was adjusted to cover its large uncertainty during the entire prediction period. Vaccine protection against HBV infection, which in newborns is closely related with timeliness and quality of administration and combination with HBIG, was estimated at 95% (92% to 98%) according to results of several classic meta-analyses with or without HBIG as an additional intervention [16–18]. The protection obtained from vaccine or infection was considered lifetime [19]. The remaining parameters were estimated from published literature. Model parameters were summarized in Table 1. A relatively wide range was given to each parameter to cover the majority of reported data.

**Table 1:** Estimates of parameters used in the model

Parameter	Base-case value	Range	Distribution	References
*λ*_*a,t*_, age- and time-dependent force of HBV infection	Table S1 and Figure S2: base-case sets	Table S1 and Figure S2: 95% confidence interval sets	Uniform	
*q*_*a*_, age-dependent proportion of acute HBV infections that become chronic				[20]
< 1 year	0.3	± 20%	Uniform (0.24, 0.36) Uniform (0.2, 0.3) Uniform (0.048, 0.072) Uniform (0.032, 0.048)	
1 − 5 years	0.25			
6 − 19 years	0.06			
≥ 20 years	0.04			
*r*, rate of chronic HBV infections that become immune (HBV clearance)	0.01	0.005–0.02	Triangular (0.005, 0.01, 0.02)	[21, 22]
*d*_*Aa*_, age-dependent mortality of acute HBV infection				Based on the age-specific risks of symptomatic infection and fulminant hepatitis and the fatality rate of fulminant hepatitis [23, 24]
< 1 year	0.000007	± 50%	Uniform (0.0000035, 0.0000105)	
1 − 5 years	0.00042		Uniform (0.00021, 0.00063)	
≥ 6 years	0.00126		Uniform (0.00063, 0.00189)	
*d*_*Ca*_, age-dependent mortality of cirrhosis	Age-specific HBV-related cirrhosis mortality curve	± 50%	Uniform	[23]
*d*_*Ha*_, age-dependent mortality of hepatocellular carcinoma	Age-specific HBV-related hepatocellular carcinoma mortality curve	± 50%	Uniform	[23]
*v*_*t*_, vaccine coverage of newborns in 2006 and beyond	0.94	0.9 − 0.98	Normal (0.94, 0.020408)	[12–15]
*p*_*t*_, vaccine protection against HBV infection in 2006 and beyond^a^	0.95	0.92 − 0.98	Normal (0.95, 0.015306)	[16–18]
*θ*_*a,t*_, catch-up vaccine coverage during 2009 − 2011 in children born between 1994 and 2001 who missed the routine vaccination	0.95	0.9 − 0.97	Uniform (0.9, 0.97)	[25]
*ε*, HBV intrauterine infection rate in infected pregnant women	0.03	0.02 − 0.035	Triangular (0.02, 0.03, 0.035)	[26–28]

^a^Also be used in the catch-up vaccination during 2009 − 2011

### Prediction analysis

Matlab.2015b (The MathWorks, Inc.) was used for modelling. Predictions were made under three scenarios.

First, we used the model to generate predictions on the prevalence under the assumption that current interventions remain at status quo levels (status quo). Following base-case analysis, Monte Carlo probabilistic sensitivity analysis was done to examine potential impacts of all parameter uncertainties. In the probabilistic sensitivity analysis, parameters with uncertainty were sampled from their respective distributions in each iteration, and for those considered to be age-dependent a positive correlation was set between age groups to avoid violating their known relationships with age. Choices of distributions were based on the consideration of properties of the parameters and data informing the parameters. In addition, one-way sensitivity analysis was done to identify sensitive parameters, in which each parameter was adjusted independently in their respective ranges.

Second, according to the WHO’s updated guideline in 2020 for prevention of HBV mother-to-child transmission [29], we assumed that peripartum antiviral prophylaxis (PAP) as a new additional intervention was introduced into the status quo scenario in 2021 and the coverage of successful interruption in mothers with high viral load increased linearly to a certain level (referring to the 90% protection reported by a most recent meta-analysis [30]) by 2030 (status quo + PAP). This was realized by adding the successful interruption coverage to the model to control HBV intrauterine infection rate. Inputs of all the model parameters were set to the base-case values.

Third, we further predicted the impact of the combination of scaling up current interventions and PAP on the prevalence (scaling up current interventions + PAP). In this scenario, the assumption of introducing PAP as above was retained and, meanwhile, sensitive intervention parameters identified by the above one-way sensitivity analysis were adjusted linearly from their respective base-case values in 2021 to the optimum (maximal or minimal) limits of their respective ranges by 2030. The other parameters were fixed at the baseline values.

## Results

### HBsAg prevalence under status quo scenario

**Fig. 2:**
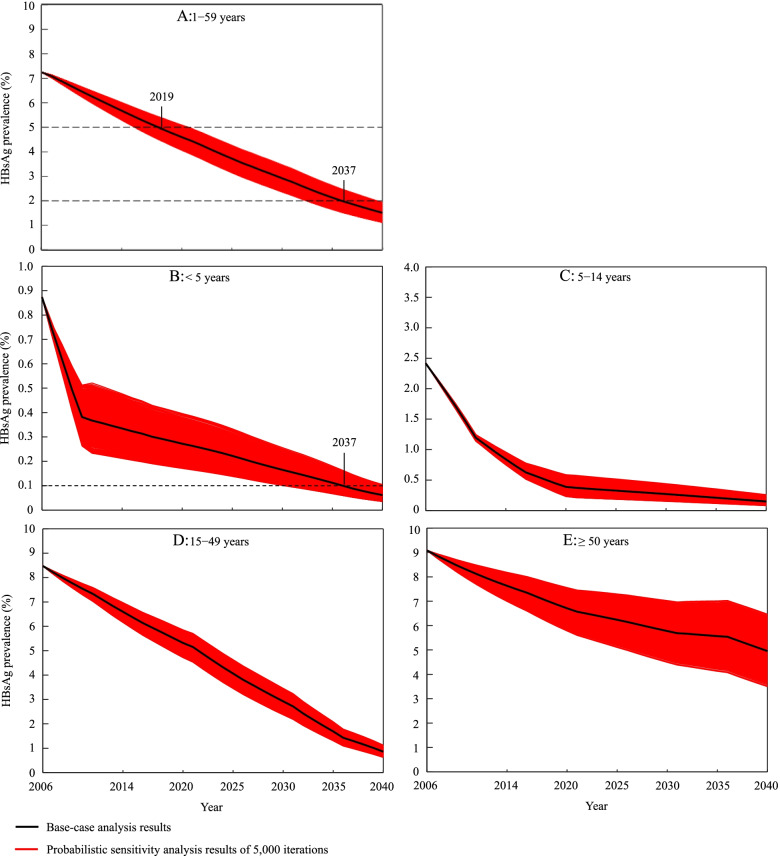
HBsAg prevalence with time in different age groups under status quo scenario

Figure 2 showed our base-case analysis and probabilistic sensitivity analysis results under the status quo scenario. As expected, HBsAg prevalence would decrease steadily from 2006 to 2040 not only in younger age groups but in the elderly aged ≥ 50 years. For the population aged 1 − 59 years (Fig. 2A), it would drop to 5% in 2019 and 2% in 2037 under base-case values. For the children aged < 5 years (Fig. 2B), however, the WHO’s 0.1% target would not be achieved until 2037 under base-case values, and the probability of meeting this target before 2036 was less than 5% according to the probabilistic sensitivity analysis.

**Fig. 3:**
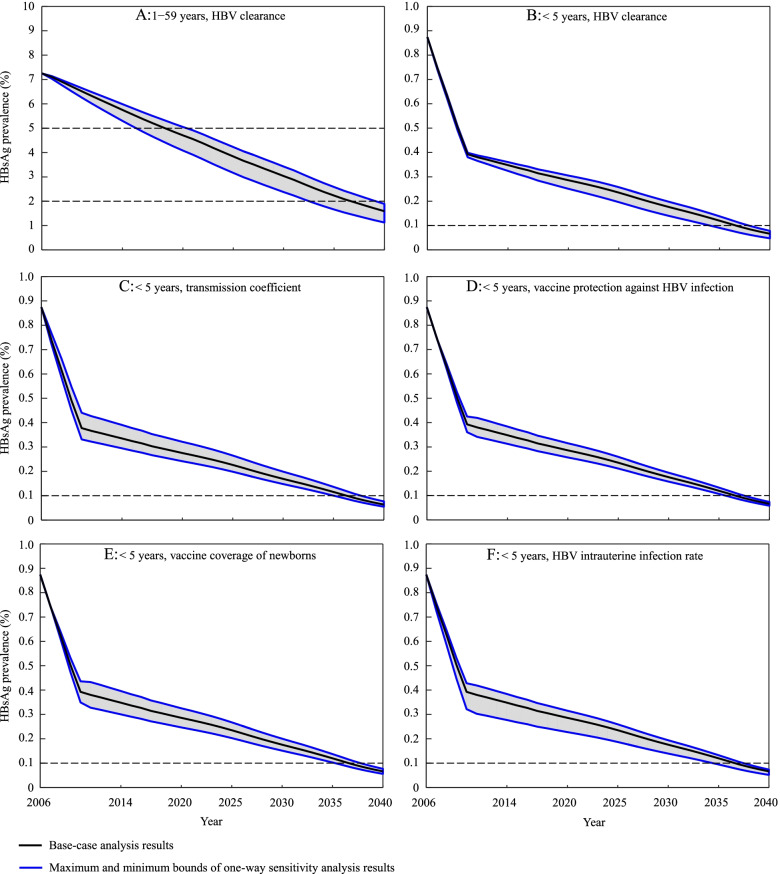
Impacts of sensitive parameters on HBsAg prevalence in populate aged 1 − 59 years and children aged < 5 years under status quo scenario

One-way sensitivity analyses showed that for the population aged 1 − 59 years, HBV clearance was the only sensitive parameter that significantly influenced the decline of HBsAg prevalence from 2006 to 2040 when it changed within the pre-set range (Fig. 3A). For children aged < 5 years, a few sensitive parameters were found, including HBV clearance, the transmission coefficient, vaccine protection against HBV infection, vaccine coverage of newborns, and HBV intrauterine infection rate. Their impacts were shown in Fig. 3B − F, sorted from large to small according to their corresponding time span size to achieve the WHO’s 0.1% target. However, no single parameter change within its pre-set range would reduce the prevalence to 0.1% by 2034. A two-way sensitivity analysis of vaccine coverage of newborns and vaccine protection against HBV infection was also done given their importance and uncertainty, and the results found that the WHO’s 0.1% target could only be achieved between 2033 − 2034 when the two parameters reached 98% simultaneously (Figure S5 of Supplementary Materials).

### HBsAg prevalence under status quo + PAP

**Fig. 4:**
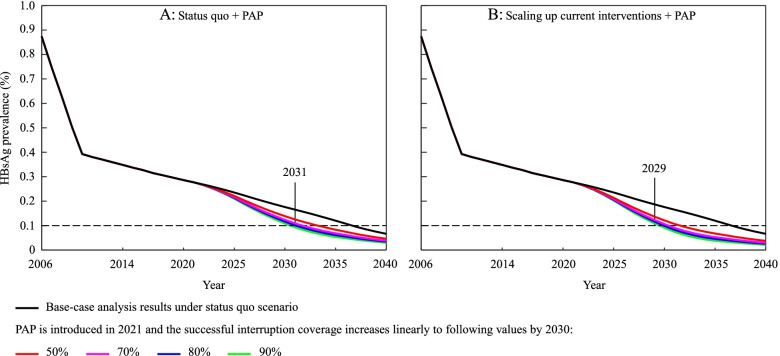
HBsAg prevalence with time in children aged < 5 years under status quo + PAP scenario and scaling up current interventions + PAP scenario, respectively. PAP, peripartum antiviral prophylaxis

Under the status quo + PAP scenario, the HBsAg prevalence of children aged < 5 years would significantly decrease with the introduction and scale-up of PAP, and the higher the successful interruption coverage is achieved, the more significant the decline (Fig. 4A). However, even if the successful interruption coverage reaches 90% by 2030, the 0.1% prevalence target would not be met until 2031.

### HBsAg prevalence under scaling up current interventions + PAP

In the scaling up current interventions + PAP scenario, the three sensitive intervention parameters including the transmission coefficient, vaccine protection against HBV infection, and vaccine coverage of newborns were adjusted simultaneously to express scale-up of current interventions. HBV clearance was not considered because no corresponding intervention is available so far. As shown in Fig. 4B, combined with scale-up of current interventions, the WHO’s 0.1% target would be achieved on time or one year in advance if PAP is introduced and the successful interruption coverage is scaled up to 80% or 90% by 2030, respectively.

## Discussion

In China and other highly HBV-endemic countries, mother-to-child transmission is the most important mode of HBV, and the prevention of mother-to-child transmission is the key to reduce new chronic HBV infections and control hepatitis B prevalence [29]. Since the introduction of hepatitis B vaccine, the Chinese government has taken a number of measures to increase the full three-dose vaccine coverage and the birth dose coverage in children and, as the result, the high coverage of > 90% had been achieved for both in the early and late 2000s, respectively [7]. HBIG as a supplement has also been used early in China for newborns born to HBsAg-positive mothers, and it was introduced into the national program integrating prevention of mother-to-child transmission of human immunodeficiency virus, syphilis, and HBV in 2012 [31]. With economic and medical development, the Chinese government adopted increasingly interventions to control hepatitis B [7], such as safe injection practice, blood donation screening, management and treatment of chronic HBV-infected persons, and even extensive health education. These non-immunization interventions can reduce risk contacts and even the probability of transmission following a risk contact, and their combined effects were integrated into our model by the transmission coefficient. There is no doubt that HBsAg prevalence will continue to decline steadily in China by maintaining current interventions, not only in children but also in the elderly, as predicted by our model. However, it is difficult for China to achieve the WHO’s target of 0.1% prevalence in children by 2030, if only current interventions are maintained. The results were robust according to our sensitivity analyses.

A small number of newborns are still infected with HBV despite the birth dose vaccine and HBIG. The failure occurs mostly in newborns born to mothers with high viral load, as a result of intrauterine infections [32]. Increasing evidences demonstrated that the use of antivirals in late pregnancy can interrupt this type of vertical transmission by 90%, and that the safety is acceptable [30]. Based on this, the WHO updated its guideline in 2020, to recommend antiviral prophylaxis as an additional measure in eligible pregnant women for preventing HBV mother-to-child transmission and achieving the target of eliminating hepatitis B [29]. An expert consensus on how to use PAP has been reached recently in the Chinese medical community [33]. The routine antenatal test for HBV markers has earlier been implemented for all pregnant women in China. With the sharp decline in antiviral drug prices in recent years in China, hepatitis B antiviral therapy has been included in the national healthcare insurance. These ensure that PAP can be introduced and generalized as soon as possible. Our model predicted that PAP would play an important role to reduce HBsAg prevalence in children and achieve the WHO’s target by 2030. It may help China significantly shorten the period to meet the target of 0.1% prevalence in children if the successful interruption coverage is steadily scaled up. This finding is important for countries where mother-to-child transmission is the main mode of HBV and the birth dose vaccine coverage (including HBIG for eligible newborns) and the full vaccine series coverage for children have reached a high level. However, this innovation alone is not sufficient for China to achieve the target on schedule. A comprehensive scale-up of available interventions, including current immunization and non-immunization measures and, especially, innovations like PAP, is needed for China to achieve the WHO’s target of 0.1% prevalence in children by 2030. Although there is a limited space for China to further expand immunization intervention coverages in children, unremitting efforts are needed, especially in rural areas, because a high HBsAg prevalence of 5.76% is still held by women of childbearing age in China [34], and vaccination starting at birth is the foundation of preventing HBV mother-to-child transmission [29].

China has a large number of chronic HBV carriers, which may maintain the virus circulation and a high HBsAg prevalence in the whole population for a long time. Natural HBV clearance is difficult in chronic infections, with an annual probability of around 1% [20]. Current antivirals provide an opportunity that can keep HBV under control, slow the progression of cirrhosis, reduce incidence of liver cancer and improve long term survival, but it is not a cure because it cannot completely clear HBV from infected cells [35]. Therefore, the treatment of patients with chronic hepatitis B played a very limited role in reducing HBsAg prevalence until now. Our model found that improving HBV clearance by treatment would be the most important factor to reduce HBsAg prevalence not only in the whole population but in children. However, the innovation is on the way.

There are three main limitations in our study. First, the vaccination in adults was not considered in the model, due to the lack of data, which may lead to an estimate of longer time to achieve the target. However, this bias should be small, because adult hepatitis B vaccination in China follows the policy of “self-select and self-pay” and infected adults rarely develop chronic infection. Second, age-dependent mortalities of cirrhosis and hepatocellular carcinoma come from an international modelling study [23], which may be different from China. We adjusted the data by ± 50%, hoping to cover the situation in China. One-way sensitivity analyses found that changes of these two parameters have only a very small impact in the elderly and almost no impact in younger age groups. Third, our study did not focus on the cost-effectiveness of various possible intervention strategies, which limits policy-makers to make clearer choices and judgments. The further studies are needed.

## Conclusions

Our model predicted that it is difficult for China to achieve the WHO’s target of 0.1% HBsAg prevalence in children by 2030 by maintaining current interventions, although HBsAg prevalence will continue to decline steadily in the whole population. PAP may play an important role to shorten the time to achieve the target. A comprehensive scale-up of available interventions including PAP will ensure that China achieves the target on schedule.

## Supplementary Information

**Additional file 1.**

## Data Availability

All data generated or analysed during this study are included in this published article and its supplementary information files.

## Abbreviations

WHO: World Health Organization
HBV: Hepatitis B virus
HBsAg: Hepatitis B virus surface antigen
PAP: Peripartum antiviral prophylaxis
HBIG: Hepatitis B immunoglobulin
