# Supplementary material for: Modelling the prevalence of hepatitis B towards eliminating it as a major public health threat in China
Source: BMC Public Health. 2022 Jun 13;22:1179. doi: 10.1186/s12889-022-13594-y (PMC9195287; doi:10.1186/s12889-022-13594-y)
Supplement: Supplementary file 1 — Additional file 1. [file 12889_2022_13594_MOESM1_ESM.docx]

**Supplementary Materials**

**The age- and time-dependent force of HBV infection**

We estimated the age- and time-dependent force of HBV infection (*λ_a,t_*) based on the previous national hepatitis B serosurvey data by following steps.

First, we estimated the age-dependent force of HBV infection in 1992 (*λ_a,1992_*) from the 1992 serosurvey data by a modified simple catalytic model. The serosurvey in 1992 was performed just before the recommendation of routine hepatitis B vaccination for newborns in China, and thus the data can characterize natural HBV infections without vaccination. In addition, the total and age-specific prevalence of HBV infection in 1992 were consistent with the results of the 1979 serosurvey [1]. For details, please see our previous published literature [2,3]. Through this step, we obtained a set of estimates of *λ_a,1992_* for five age groups (Table S1), including base-case values and 95% confidence intervals (CIs). The division of these five age groups is based on the transmission mode and characteristics of HBV between populations with different ages. For example, 15−49 years old correspond to the ages of women of childbearing age.

Table S1 Estimates of the age-dependent force of HBV infection in 1992 for the five age groups

| Age group (years) | *λ_a,1992_* | Base-case value | 95% CI |
| --- | --- | --- | --- |
| 0 | *λ_0,1992_* | 0.190608 | 0.129501−0.268389 |
| 1−4 | *λ_1−4,1992_* | 0.058402 | 0.032190−0.108758 |
| 5−14 | *λ_5−14,1992_* | 0.014083 | 0.011470−0.020531 |
| 15−49 | *λ_15−49,1992_* | 0.013202 | 0.011334−0.015071 |
| ≥50 | *λ_≥50,1992_* | 0.013202 | 0.011334−0.015070 |

Second, we built a 5 × 5 matrix of “who acquires infection from whom” (WAIFW) to characterize HBV transmission relationships between the five age groups (Figure S1), in which five transmission coefficients (*φ_1_*, *φ_2_*, *φ_3_*, *φ_4_*, and *φ_5_*) need to be solved. We modified the theoretical function of the force of infection Equation (3) to Equation (S1) according to the matrix, and by Equation (S1) we obtained estimates of the five transmission coefficients in 1992 (Table S2) from *λ_a,1992_* and the relevant population data in 1992. Please refer to relevant literature for the method of WAIFW matrix [4-6].

|  |  | Age of infectious individuals (years) | | | | |
| --- | --- | --- | --- | --- | --- | --- |
|  |  | 0 | 1−4 | 5−14 | 15−49 | ≥50 |
| Age of susceptible individuals (years) | 0 | *φ*_5_ | *φ*_5_ | *φ*_5_ | *φ_1_* | *φ*_5_ |
|  | 1−4 | *φ*_5_ | *φ*_5_ | *φ*_5_ | *φ*_2_ | *φ*_5_ |
|  | 5−14 | *φ*_5_ | *φ*_5_ | *φ*_5_ | *φ*_3_ | *φ*_5_ |
|  | 15−49 | *φ*_5_ | *φ*_5_ | *φ*_5_ | *φ*_4_ | *φ*_5_ |
|  | ≥50 | *φ*_5_ | *φ*_5_ | *φ*_5_ | *φ*_5_ | *φ*_5_ |

Figure S1 Matrix of WAIFW of HBV between the five age groups

$\left\{\begin{matrix} \lambda_{0}=\phi_{1}\frac{C_{15\text{-}49}}{N_{15\text{-}49}}+\phi_{5}\left(\frac{C_{0}}{N_{0}}+\frac{C_{1\text{-}4}}{N_{1\text{-}4}}+\frac{C_{5\text{-}14}}{N_{5\text{-}14}}+\frac{C_{\geq50}}{N_{\geq50}}\right) \\ \lambda_{1\text{-}4}=\phi_{2}\frac{C_{15\text{-}49}}{N_{15\text{-}49}}+\phi_{5}\left(\frac{C_{0}}{N_{0}}+\frac{C_{1\text{-}4}}{N_{1\text{-}4}}+\frac{C_{5\text{-}14}}{N_{5\text{-}14}}+\frac{C_{\geq50}}{N_{\geq50}}\right) \\ \lambda_{5\text{-}14}=\phi_{3}\frac{C_{15\text{-}49}}{N_{15\text{-}49}}+\phi_{5}\left(\frac{C_{0}}{N_{0}}+\frac{C_{1\text{-}4}}{N_{1\text{-}4}}+\frac{C_{5\text{-}14}}{N_{5\text{-}14}}+\frac{C_{\geq50}}{N_{\geq50}}\right) \\ \lambda_{15\text{-}49}=\phi_{4}\frac{C_{15\text{-}49}}{N_{15\text{-}49}}+\phi_{5}\left(\frac{C_{0}}{N_{0}}+\frac{C_{1\text{-}4}}{N_{1\text{-}4}}+\frac{C_{5\text{-}14}}{N_{5\text{-}14}}+\frac{C_{\geq50}}{N_{\geq50}}\right) \\ \lambda_{\geq50}=\phi_{5}\left(\frac{C_{0}}{N_{0}}+\frac{C_{1\text{-}4}}{N_{1\text{-}4}}+\frac{C_{5\text{-}14}}{N_{5\text{-}14}}+\frac{C_{15\text{-}49}}{N_{15\text{-}49}}+\frac{C_{\geq50}}{N_{\geq50}}\right) \end{matrix}\right.$ (S1)

Table S2 Estimates of the transmission coefficient in 1992 between the five age groups

| *φ* | Base-case value | 95% CI |
| --- | --- | --- |
| *φ_1_* | 1.860781 | 1.246224−2.646920 |
| *φ_2_* | 0.500391 | 0.244906−1.004327 |
| *φ_3_* | 0.044355 | 0.031695−0.096473 |
| *φ_4_* | 0.035291 | 0.030297−0.040290 |
| *φ_5_* | 0.035291 | 0.030297−0.040284 |

Third, we selected an exponential function to characterize the decline of all the five transmission coefficients from 1992 to 2006 (Equation (S2)), according to published literature [7,8]. The decline can be attributed to the decrease in risk contact, and may also be attributed to the decrease in probability of transmission following a risk contact in which antiviral treatment of patients with chronic hepatitis B can be one of the reasons.

$\varphi_{a,t+1}=[m\exp\left( nt \right)+r]\varphi_{a,t}$ (S2)

Where *m* = (0, 1), *n* = (-1, 0), *r* = (0, 1) and *m* + *r* = 1.

Parameters *m*, *n* and *r* of Equation (S2) for each transmission coefficient, as a set, were estimated by Markov Chain Monte Carlo (MCMC) method with a Metropolis-Hastings (M-H) algorithm [9-11]. Prior distributions of these parameters were assumed to be uniform, and they were sampled from their respective ranges as inputs to enter our model (Figure 1). The model was run from 1992 to 2006 for 10,000 iterations to gradually converge the inputs of these parameters and obtain their posteriori normal distributions [12,13], in which the 2006 serosurvey data were used as calibrations of the model outputs [14]. Means of the posteriori normal distributions were selected to form the most appropriate set of these parameters. In this process, the base-case values of the five transmission coefficients in 1992 and their upper and lower limits of 95% CIs were respectively introduced into the model as a set, in order to obtain the corresponding most appropriate estimates of base-case set and two 95% CI sets of these parameters. Inputs of the two model parameters, vaccine coverage of newborns (*v_t_*) and vaccine protection against HBV infection (*p_t_*), were considered to increase annually from 1992 to 2006, as done in our previous study [2]. The consideration of the annual increase of vaccine protection against HBV infection from 1992 to 2006 is mainly because during this period, the birth dose vaccine coverage increased year by year. Inputs of the other model parameters were fixed at their respective base-case values, as showed in Table 1. Decline curves of the five transmission coefficients from 1992 to 2006 were showed in Figure S2, and the comparison of model outputs in 2006 with the 2006 serosurvey data was showed in Figure S3.

Finally, the model was further run from 2006 to 2014, in which the same declining pattern of the transmission coefficients was maintained but new initial conditions of the model were introduced based on the 2006 serosurvey data and China Population and Employment Statistics Yearbook, 2007. The model outputs in 2014 were compared with the 2014 serosurvey data [15], in order to validate our model (Figure S4).


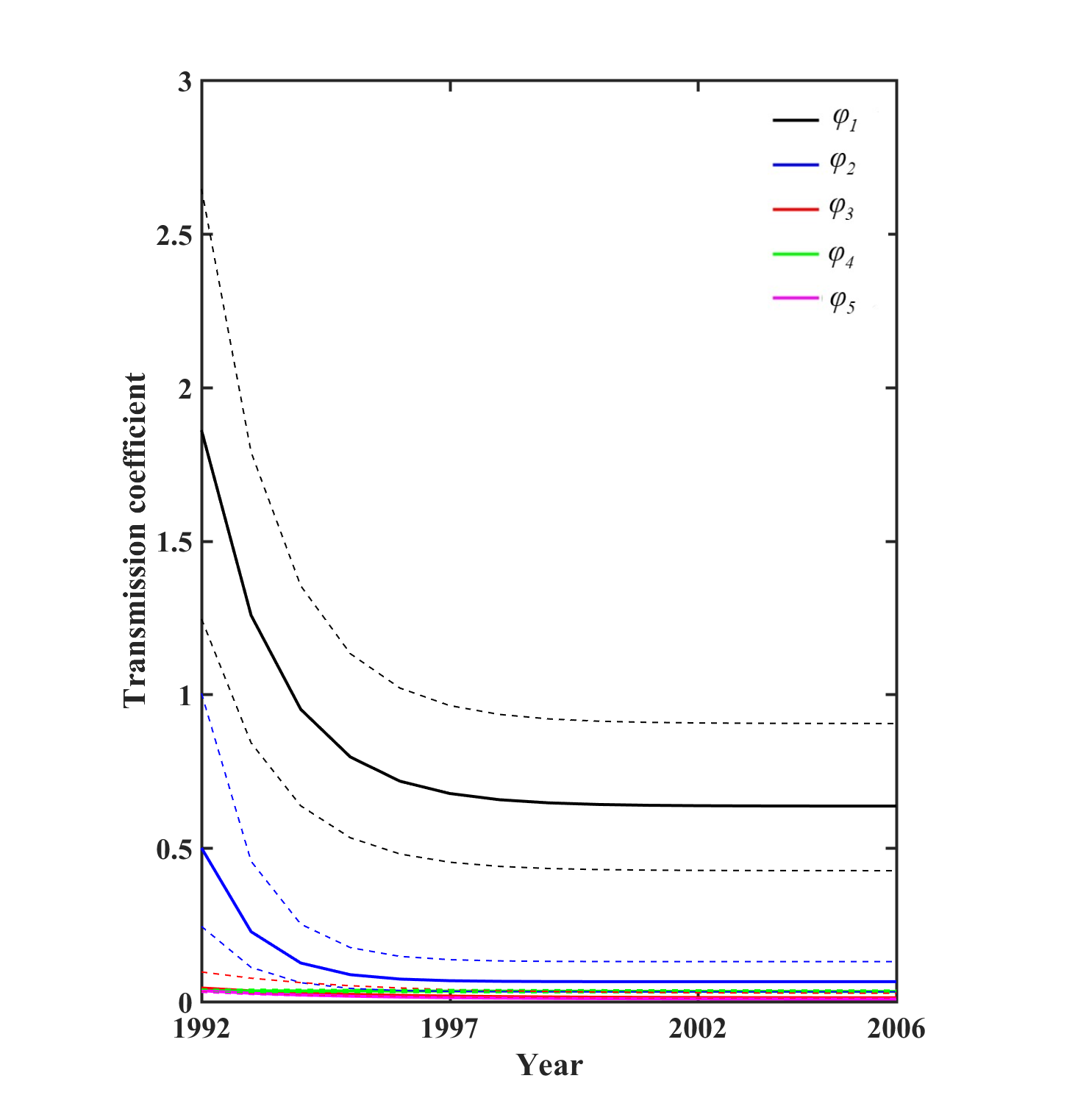


Figure S2 Base-case values and 95% CIs of the five transmission coefficients from 1992 to 2006


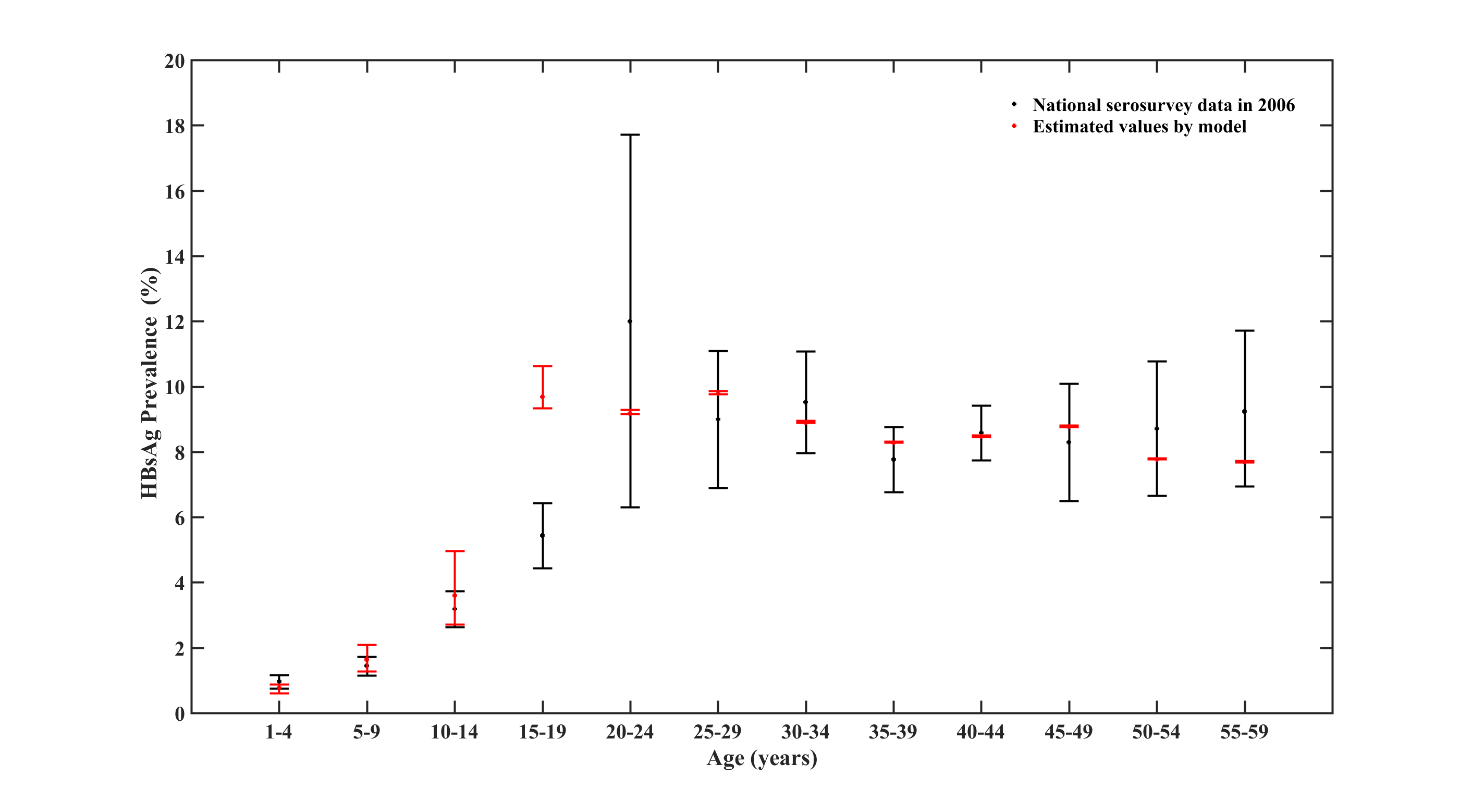


Figure S3 HBsAg prevalence (point estimate and 95% CI) in different age groups in 2006

**
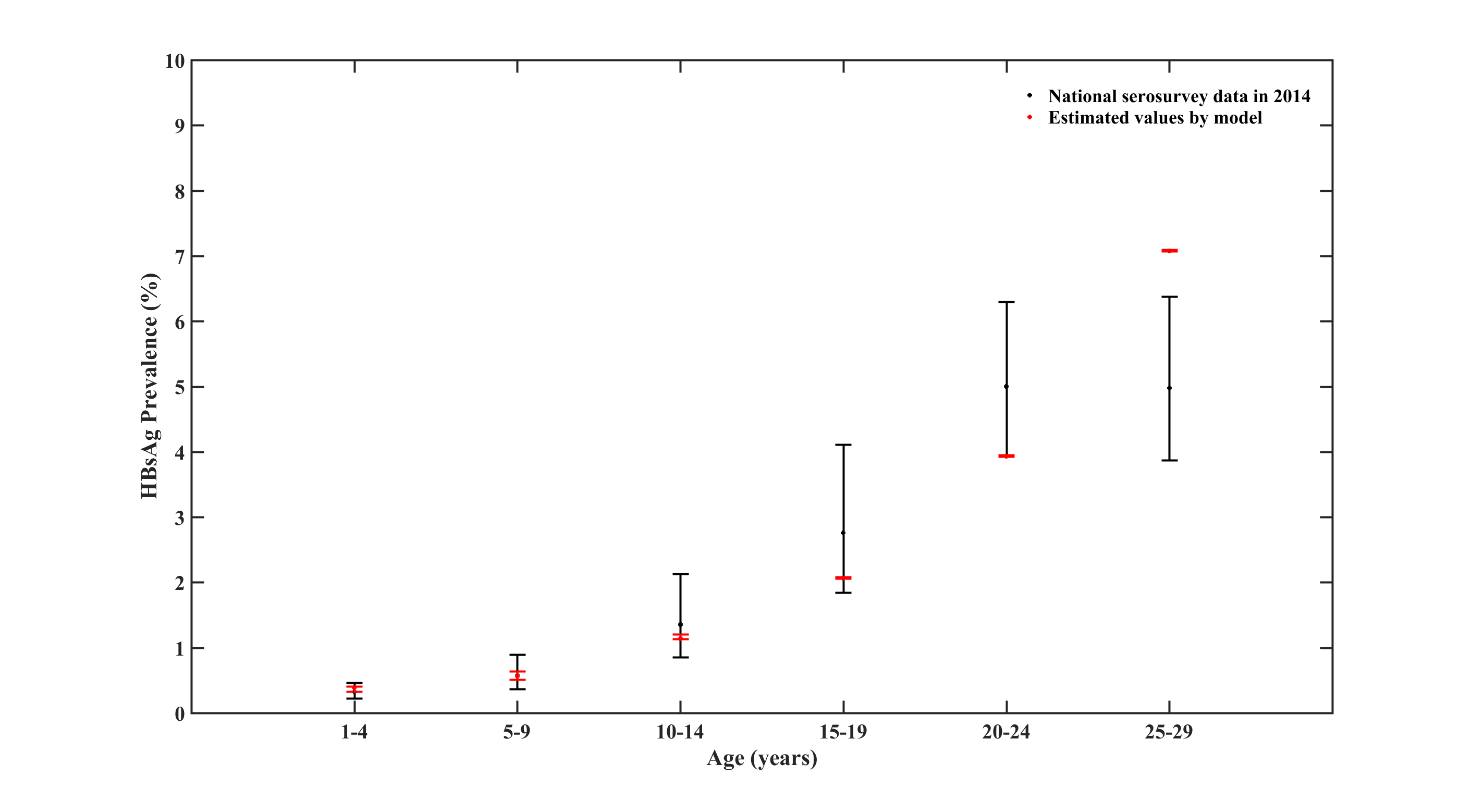
**

Figure S4 HBsAg prevalence (point estimate and 95% CI) in different age groups in 2014

**The two-way sensitivity analysis results of vaccine coverage of newborns and vaccine protection against HBV infection**

2006

2014

2020

2025

2030

2035

2040

0

0.1

0.2

0.4

0.5

0.6

0.7

0.8

0.9

1.0

0.3

HBsAg prevalence (%)

Year

Base-case analysis results

Results if both vaccine coverage of newborns and vaccine protection against HBV infection reach 98% simultaneously

Figure S5 Impacts of the combination of both vaccine coverage of newborns and vaccine protection against HBV infection on HBsAg prevalence in children aged < 5 years under status quo scenario

**References**

1. Dai Z, Qi G. Viral hepatitis in China–seroepidemiological survey in Chinese population, 1992−1995 (part one). Beijing: Scientific and Technical Documents Publishing House; 1999 (Chinese).

2. Liang P, Zu J, Yin J,Li H, Gao L, Cui F, et al. The independent impact of newborn hepatitis B vaccination on reducing HBV prevalence in China, 1992−2006: A mathematical model analysis. J Theor Biol. 2015;386:115-121.

3. Yin J, Ji Z, Liang P, Wu Q, Cui F, Wang F, et al. The doses of 10 μg should replace the doses of 5 μg in newborn hepatitis B vaccination in China: A cost-effectiveness analysis. Vaccine. 2015;33(31):3731-3738.

4. Anderson RM, May RM. Age-related changes in the rate of disease transmission: implications for the design of vaccination programmes. J Hyg (Lond). 1985;94(3):365-436.

5. Read JM, Lessler J, Riley S, Wang S, Tan LJ, Kwok KO, et al. Social mixing patterns in rural and urban areas of southern China. Proc Biol Sci. 2014;281(1785):20140268.

6. Harris RC, Sumner T, Knight GM, [Evans](https://pubmed.ncbi.nlm.nih.gov/?term=Evans+T&cauthor_id=30630775) T, [Cardenas](https://pubmed.ncbi.nlm.nih.gov/?term=Cardenas+V&cauthor_id=30630775) V, Chen C, et al. Age-targeted tuberculosis vaccination in China and implications for vaccine development: a modelling study. Lancet Glob Health. 2019;7(2):e209-e218.

7. Nagelkerke N, Heisterkamp S, Borgdorff M, Broekmans J, Van Houwelingen H. Semi-parametric estimation of age-time specific infection incidence from serial prevalence data. Stat Med. 1999;18(3):307-320.

8. Srinivasa Rao AS, [Chen](https://pubmed.ncbi.nlm.nih.gov/?term=Chen+MH&cauthor_id=17147828) MH, [Pham](https://pubmed.ncbi.nlm.nih.gov/?term=Pham+BZ&cauthor_id=17147828) BZ, [Tricco](https://pubmed.ncbi.nlm.nih.gov/?term=Tricco+AC&cauthor_id=17147828) AC, [Gilca](https://pubmed.ncbi.nlm.nih.gov/?term=Gilca+V&cauthor_id=17147828) V, [Duval](https://pubmed.ncbi.nlm.nih.gov/?term=Duval+B&cauthor_id=17147828) B, et al. Cohort effects in dynamic models and their impact on vaccination programmes: an example from hepatitis A. BMC Infect Dis. 2006;6:174.

9. Haario H, Laine M, Mira A, Saksman E. DRAM: Efficient adaptive MCMC. Stat Comput. 2006;16:339-354.

10. Morton A, Finkenstädt BF. Discrete time modelling of disease incidence time series by using Markov chain Monte Carlo methods. Appl Statist. 2005;54:575-594.

11. Trentini F, Poletti P, Merler S, Melegaro A. Measles immunity gaps and the progress towards elimination: a multi-country modelling analysis. Lancet Infect Dis. 2017;17(10):1089-1097.

12. Guo P, Zhu B, Niu H, Wang Z, Liang Y, Chen Y, et al. Fast genomic prediction of breeding values using parallel Markov chain Monte Carlo with convergence diagnosis. BMC Bioinformatics. 2018;19(1):3.

13. Harms RL, Roebroeck A. Robust and Fast Markov Chain Monte Carlo Sampling of Diffusion MRI Microstructure Models. Front Neuroinform. 2018;12:97.

14. Chinese Center for Disease Control and Prevention. Seroepidemiological investigation of HBV in Chinese population. Beijing: People's Medical Publishing House; 2011 (Chinese).

15. Bureau of Disease Prevention and Control, National Health Commission, Chinese Center for Disease Control and Prevention. The national report of hepatitis B serosurvey among 1−29 years old population in China. Beijing: People's Medical Publishing House; 2018 (Chinese).
